# Postprandial response of plasma IL-6 to isoenergetic meals rich in casein or potato singly and combined in obese women

**DOI:** 10.1017/jns.2013.25

**Published:** 2013-09-06

**Authors:** Patrick J. Manning, Wayne H. F. Sutherland, Sylvia A. de Jong, Anne R. Ryalls, Elizabeth A. Berry

**Affiliations:** Department of Medicine, Dunedin School of Medicine, University of Otago, Dunedin, New Zealand

**Keywords:** IL-6, Obesity, Casein, Carbohydrate, Postprandial responses, CA, casein, POT, potato, POT + CA, potato + casein.

## Abstract

Milk consumption decreases inflammatory stress in overweight and obese subjects. Casein is the major protein in milk and enhances the secretion of insulin that has anti-inflammatory activity. The aim of the present study was to compare the acute effect of meals rich in casein and carbohydrate and a combination of both nutrients on postprandial plasma concentrations of IL-6, a marker of inflammation, in obese women. A total of twenty-five obese women aged 38–68 years consumed isoenergetic meals rich in potato (POT) or casein (CA) or a combination of both these meals (POT + CA), in random order in a cross-over trial. After an overnight fast, blood samples were collected before and at 1 and 4 h after the meals and circulating concentrations of IL-6, glucose, insulin and NEFA were measured. Plasma IL-6 concentrations increased significantly (*P* < 0·001) during 4 h after the meals. The AUC of postprandial IL-6 concentrations was not significantly (*P* = 0·77) different among the meals. Postprandial serum insulin concentration AUC was significantly higher during the POT + CA meal compared with the POT meal (*P* = 0·001) and the CA meal (*P* < 0·05), which in turn was significantly higher than the POT meal (*P* < 0·05). These data show that while ingestion of CA alone or combined with POT acutely increases circulating insulin concentrations, it does not appreciably alter the postprandial increase in plasma IL-6 concentrations in obese women.

IL-6 is a multifunctional cytokine that is synthesised in several tissues, and regulates innate immunity, the acute-phase response and central and peripheral nutrient homeostasis^(^^1^^)^. Under non-inflammatory conditions, adipose tissue supplies approximately 30 % of circulating IL-6^(^^2^^)^. In obese individuals, concentrations of IL-6 in fasting plasma^(^^3^^,^^4^^)^ and adipose tissue^(^^3^^)^ and release of IL-6 from adipose tissue into the circulation^(^^2^^)^ are abnormally high. These elevated levels of IL-6 are thought to reflect the chronic, subclinical inflammation that is associated with obesity as a result of increased numbers of macrophages in adipose tissue^(^^5^^)^. Ingestion of food acutely increases IL-6 levels in adipose tissue^(^^6^^)^ and plasma^(^^7^^–^^10^^)^ and increases the release of IL-6 from skeletal muscle^(^^11^^)^.

Milk products are an important source of protein in the Western diet. Consumption of low-fat dairy products is inversely associated with the risk of developing type 2 diabetes^(^^12^^)^. Casein accounts for 80 % of milk proteins and diets rich in casein seem to decrease body weight in obese women^(^^13^^)^. An increase in milk intake for 28 d decreases fasting plasma IL-6 concentrations in overweight and obese individuals^(^^14^^)^. Ingestion of casein, like other proteins, enhances the secretion of insulin that is known to inhibit inflammation^(^^15^^,^^16^^)^. Few if any studies have examined the acute effect of consuming meals rich in casein and casein plus carbohydrate on postprandial plasma IL-6 concentrations in obese subjects.

The aim of the present study was to compare the acute effects of isoenergetic meals containing casein or carbohydrate or in combination on plasma IL-6 concentrations in obese women.

## Subjects and methods

### Subjects

A total of twenty-five women with BMI ≥ 30 kg/m^2^ and aged 38–68 years, including eleven who did not have serious illnesses and were not receiving any medications and fourteen who were receiving prescribed medications for hypertension (*n* 8) and depression (*n* 8), were recruited. The present study was conducted according to the guidelines laid down in the Declaration of Helsinki and all procedures involving human subjects/patients were approved by the Lower South Regional Ethics Committee. Written and informed consent was obtained from all participants.

### Study design

The study had a single-blind, randomised, cross-over design. Participants were randomly assigned to a sequence of three test meals using the second generator on the www.randomization.com website. There was at least 1 week between each meal. After an overnight fast, participants reported to the study centre in the early morning (08.00 hours). A venous blood sample was taken by venepuncture and a meal was immediately consumed within 15 min. Further blood samples were then taken at 1 and 4 h after the meals. Participants were allowed to drink water but not other beverages and food and they remained seated during the study. Participants were instructed to maintain their usual lifestyle in the periods between the meals.

### Meals

The potato (POT) meal contained 20 g dried potato flakes (Cinderella) that was reconstituted into mashed potato by the addition of hot water (80 ml). The casein (CA) meal contained 19·5 g of sodium caseinate (Fonterra), 2·5 g cocoa powder and 1·5 teaspoons of saccharin dissolved in 150 ml water. Consumption of both the POT meal and the CA meal at the same time constituted the POT + CA meal. The composition of meals is shown in Table 1.

**Table 1.** Composition of the meals

Meal…	POT	CA	POT + CA
Energy (kJ)	309	308	617
Carbohydrate (g)	18·0	0·05	18·0
Protein (g)	1·3	18·0	19·0
Fat (g)	0·1	0·15	0·25

POT, potato; CA, casein; POT + CA, potato + casein.

### Laboratory methods

Venous blood was taken into tubes containing EDTA, fluoride and into plain tubes. Serum and EDTA plasma were separated by centrifugation of the tubes at 1500 ***g*** for 15 min at 4°C. Samples of serum and plasma were harvested and stored at –80°C. Plasma glucose was measured in fluoride anti-coagulated blood by routine automated methods in the laboratories of Dunedin Public Hospital. Serum insulin was measured on a Hitachi 911 autoanalyser using a commercial kit and calibrator (Roche Diagnostics). Plasma IL-6 concentrations were measured in duplicate by sensitive enzyme-linked immunosorbent assay using a commercial kit (R&D Systems). The intra-assay CV for this assay was 7 %. Samples from an individual were measured in the same assay to reduce inter-assay variation.

### Statistical analyses

Data are presented as mean values and standard deviations unless stated otherwise. Data were log-transformed before statistical analysis using the IBM SPSS statistical software, version 20 (IBM Corp.). The trapezium method was used to calculate AUC^(^^17^^)^. Repeated-measures ANOVA with simple within-subject contrasts was used to compare AUC among the meals. Models were also tested with medication status as a between-subjects factor. Repeated-measures ANOVA was also used to analyse changes in variables with time after meals and to estimate carry-over by comparing zero-time values among the three visits. Two-sided tests of significance were used and a *P* value of less than 0·05 was considered to be statistically significant.

## Results

The characteristics of the obese women who participated in the study are shown in Table 2. Baseline concentrations of IL-6, glucose, insulin and NEFA in the circulation were higher compared with values (IL-6: 1·4 (sd 0·6) ng/l; glucose: 4·6 (sd 0·40) mmol/l; insulin: 27 (sd 10) pmol/l; NEFA: 0·41 (sd 0·19) mmol/l) in fourteen lean women of comparable age (53 (sd 10) years) who participated in a previous study from this laboratory^(^^9^^)^.

**Table 2.** Baseline characteristics of the participants (*n* 25) determined at the first visit (Mean values and standard deviations)

Characteristic	Mean	sd
Age (years)	54	9
BMI (kg/m^2^)	35·9	5·0
Waist (cm)	109	10
Glucose (mmol/l)	5·5	0·4
Insulin (pmol/l)	77	60
HOMA-IR	1·45	1·11
NEFA (mmol/l)	0·45	0·17
IL-6 (ng/l)	1·93	1·25

HOMA-IR, homeostatic model assessment of insulin resistance.

**Fig. 1.**
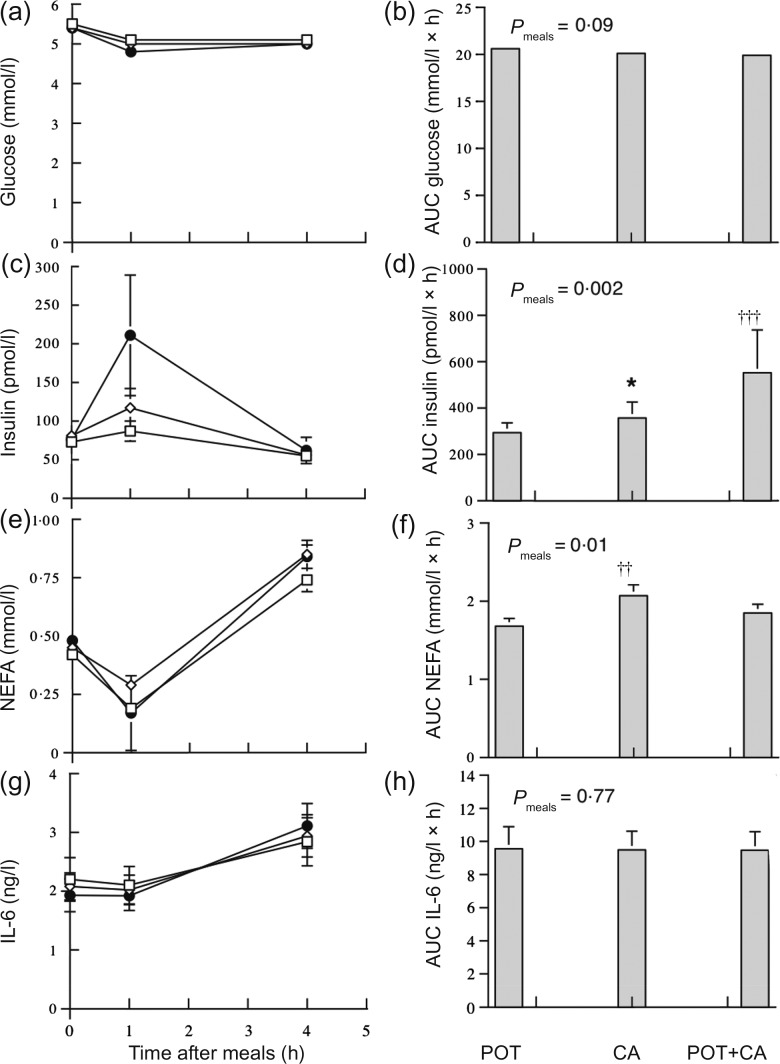
Postprandial circulating concentrations of glucose (a), insulin (c), NEFA (e) and IL-6 (g) and AUC of glucose (b), insulin (d), NEFA (f) and IL-6 (h) during meals rich in potato ( □ ; POT), casein (CAS; ◊) and potato + casein (POT + CA; ●) in obese women (*n* 25). Values are means, with standard errors represented by vertical bars. Concentrations of glucose, insulin, NEFA and IL-6 all changed significantly with time after meal (*P* < 0·001) in repeated-measures ANOVA of log-transformed data. *Mean value was significantly different from those of other meals (*P* < 0·05; within-subject contrasts in repeated-measures ANOVA of log-transformed data). Mean value was significantly different from that of the POT meal: †† *P* = 0·01, ††† *P* = 0·001 (within-subject contrasts in repeated-measures ANOVA of log-transformed data).

Fig. 1 shows postprandial circulating concentrations and AUC of glucose, insulin, NEFA and IL-6 in the obese women during the meals. Serum insulin concentrations increased significantly (*P* < 0·001) during the meals. The AUC of postprandial serum insulin concentrations was significantly (*P* < 0·05) higher during the CA meal compared with the POT meal and was significantly higher during the POT + CA meal compared with both the POT meal (*P* = 0·001) and the CA meal (*P* < 0·05). The AUC of postprandial NEFA concentrations was significantly (*P* < 0·01) higher during the CA meal compared with the POT meal. The AUC of postprandial plasma glucose and IL-6 concentrations were not significantly different among the meals. There were no significant (*P* = 0·43–0·93) interactions between medication status and type of meal in AUC data. Zero-time circulating concentrations of glucose (*P* = 0·73), insulin (*P* = 0·77), NEFA (*P* = 0·14) and IL-6 (*P* = 0·51) were not significantly different among the three visits.

## Discussion

Our data show that the postprandial increase in plasma IL-6 concentrations was similar after ingestion of isoenergetic amounts of POT and CA and a combination of both nutrients in obese women. This finding is in keeping with the results of a previous study which reported similar postprandial increases in plasma IL-6 concentrations after the consumption of mixed meals rich in protein (that was derived from soya and whey), carbohydrate, or fat in subjects with the metabolic syndrome^(^^10^^)^. Altogether, these studies suggest that the type of macronutrient and the type of protein consumed does not differentially affect the postprandial increase in plasma IL-6 concentrations.

There is evidence that insulin has anti-inflammatory activity, including a decrease in mononuclear cell NF-κB during euglycaemic hyperinsulinaemia^(^^15^^,^^16^^)^. On the other hand, an increase in plasma IL-6 concentrations at approximately 4 h during a euglycaemic–hyperinsulinaemic clamp in healthy men^(^^18^^)^, in subjects with type 2 diabetes and non-diabetic individuals^(^^19^^)^ has been reported. In the present study, postprandial serum insulin concentrations were higher after ingestion of CA and even more so after ingestion of POT + CA compared with ingestion of POT alone while the response of plasma IL-6 levels did not differ appreciably among these meals. It is possible that these postprandial increases in serum insulin concentrations were not large enough to influence plasma IL-6 concentrations. In previous studies, supraphysiological concentrations of circulating insulin were achieved during euglycaemic–hyperinsulinaemic clamps that increased plasma IL-6 concentrations^(^^18^^,^^19^^)^. A greater increase in postprandial serum insulin concentrations when casein is added to a meal has been reported previously^(^^20^^)^. Ingestion of milk and other food protein stimulate insulin secretion and increase insulin concentrations in the blood^(^^21^^,^^22^^)^.

Postprandial hyperinsulinaemia inhibits adipose tissue lipolysis and NEFA release. In the present study, postprandial NEFA response, as indicated by AUC, was unexpectedly higher following the ingestion of casein compared with potato despite a concomitantly larger increase in serum insulin concentrations after intake of casein. It is possible that gastric emptying was more rapid after the CA meal compared with the other meals and the nadir of postprandial NEFA concentrations may have been earlier than 1 h and therefore undetected. The CA meal was liquid and would be emptied more rapidly from the stomach compared with the other meals that contained solid nutrients.

The meals in the present study had low energy content. Our preliminary studies in a small number of obese women found that they were unable to comfortably consume a POT + CA meal with twice the current amounts of these nutrients. Also, the amounts of protein and carbohydrate were comparable with amounts used in previous studies^(^^21^^,^^23^^)^. Investigation of the effect of meals low in energy content in obese subjects is appropriate as they are advised to consume less food in order to lose weight.

The metabolic effect of the postprandial increase in plasma IL-6 is uncertain. There is evidence that IL-6 can affect glucose and lipid metabolism^(^^1^^)^. Recently, it has been suggested that the postprandial increase in plasma IL-6 may be due, at least in part, to enhanced skeletal muscle expression of the IL-6 gene and may be a normal, physiological response aimed at enhancing glucose uptake^(^^24^^)^.

The present study has a number of limitations. The number of subjects studied was relatively small. Thus, caution must be exercised in extrapolation of the findings to larger populations. The physical state of the meals was not identical. Thus, gastric emptying may have differed among the meals and influenced postprandial concentrations of measured variables. The proportions of carbohydrate and protein were reduced in the POT + CA meal compared with the other meals and this may alter some metabolic responses. Some of the women were taking medications. However, medication use did not appear to affect postprandial responses in our data. We did not study non-obese controls and cannot therefore directly assess the effect of obesity on our findings. The number of postprandial measurements was limited and this may also have limited the assessment of early changes in plasma insulin, glucose and NEFA after the meals. However, values of insulin and glucose at the current postprandial time points were comparable with those reported previously in healthy subjects after meals containing comparable amounts of protein and carbohydrate. After these meals, glucose concentrations were below baseline from 60 to 120 min^(^^23^^)^. Postprandial hyperinsulinaemia can increase the disposal of blood glucose so that it becomes greater than the absorption of glucose from the gut, leading to a decrease in blood glucose below baseline concentrations^(^^25^^)^.

In conclusion, these data suggest that while ingestion of CA alone or combined with POT acutely increases postprandial insulin concentrations, it does not noticeably affect the postprandial increase in plasma concentrations of IL-6.
